# Differential Resuscitative Effect of Pyruvate and its Analogues on VBNC (Viable But Non-Culturable) *Salmonella*

**DOI:** 10.1264/jsme2.ME12174

**Published:** 2013-04-17

**Authors:** Yuta Morishige, Ko Fujimori, Fumio Amano

**Affiliations:** 1 Laboratory of Biodefense & Regulation, Osaka University of Pharmaceutical Sciences, Nasahara, Takatsuki, Osaka 569–1094, Japan

**Keywords:** VBNC (viable but non-culturable), *Salmonella*, pyruvate, resuscitation, H_2_O_2_

## Abstract

An environmental isolate of *Salmonella* Enteritidis (SE), grown to the logarithmic phase, rapidly lost culturability by the addition of 3 mM H_2_O_2_ to cultures grown in Luria-Bertani (LB) medium; however, some H_2_O_2_-treated bacteria regained their culturability in M9 minimal medium, if sodium pyruvate was present at at least 0.3 mM. In addition, most pyruvate analogues, such as bromopyruvate or phenylpyruvate, did not show restoration activity similar to that of pyruvate, except in the case of α-ketobutyrate. Further analysis of the mechanism underlying the resuscitation by pyruvate revealed that although many of the bacteria showed respiratory activity on CTC (5-cyano-2,3-di-(*p*-tolyl) tetrazolium chloride) reduction with or without pyruvate, the biosynthesis of DNA and protein synthesis were quite different in the presence or absence of pyruvate, i.e., pyruvate endowed the cells with the ability to incorporate much more radio-label into precursors during the resuscitation process. These results suggest that pyruvate is one of the key molecules working in the resuscitation process by taking bacteria from the non-culturable state to the growing and colony-forming state by triggering the synthesis of macromolecules such as DNA and protein.

*Salmonella* is one of the most frequent causes of food-borne diseases in Japan, and is detected not only in food but also in natural environments, including river water, soil, manure or air-borne dust (4, 8, 16, 19, 28). Among *Salmonella* spp., *Salmonella enterica* servoar Enteritidis (SE) is the dominant species and has sometimes caused mass food poisoning in Japan (17) and the United States (4), although fatalities due to salmonellosis are low. In consideration of the strong infectivity of *Salmonella*, a better understanding and appropriate risk management of environmental *Salmonella* are necessary to control the spread of *Salmonella* to foods in general. Contamination of *Salmonella* from the environment to food is an important matter for food safety; however, *Salmonella* is known to persist and survive in the environment even after exposure to such sanitizers as hydrogen peroxide (H_2_O_2_) (1), a widely used sanitizing agent.

Since Xu *et al.* (38) reported their pioneering study concerning the existence of the viable but non-culturable (VBNC) state over 30 years ago, a large number of papers have been published by researchers worldwide, documenting the VBNC phenomenon in a wide variety of bacteria. Many pathogens, such as *Escherichia coli*, *Vibrio cholerae*, *Vibrio vulnificus*, *Shigella sonnei*, *Shigella flexneri*, *Campylobacter jejuni*, *Legionella pneumophila* (3, 5, 10, 26, 27, 32, 34, 36), and *Salmonella* Enteritidis (9) can enter the VBNC state after exposure to adverse environmental conditions such as high/low temperature, osmotic stress, oxidative stress, and nutritional starvation (2, 11, 23, 26, 37). The VBNC state is now generally accepted as a state in which a cell is metabolically active but is incapable of undergoing the cell division necessary to grow and to form a colony on growth media (21, 25, 29). In this paper, we used the definition of “culturability” as colony-forming activity of the cells on LB-agar plates, and that of “viability” primarily as metabolic activity to reduce 5-cyano-2,3-di-(*p*-tolyl) tetrazolium chloride (CTC). According to the definition, the VBNC state corresponds to the CTC-reduction-positive but colony-formation-negative state. An assay method based on the resuscitation of VBNC cells has been used to study the factors involved in reversing the VBNC state of several bacterial species. For example, *Vibrio vulnicus* and *Salmonella* Typhimurium are resuscitated after a temperature change (14, 25), and *Enterococcus* spp., when grown in tryptic soy broth (TSB; 20). In this report, we show that H_2_O_2_ exposure rapidly compelled SE cells to enter the VBNC state. Also, addition of sodium pyruvate to H_2_O_2_-treated SE cells effectively resuscitated them from the VBNC state to the growing state.

Sodium pyruvate, a well-known compound and an intermediate key metabolite of glycolysis, is known as a bacterial growth inducer for cells treated with H_2_O_2_ (6, 13, 22, 23). Although this up-regulating effect of pyruvate on resuscitation is known, the precise mechanism underlying this pyruvate-mediated promotion of growth of bacteria in the VBNC state is not yet clear.

Previously, we found that stressed *Salmonella* release a large amount of pyruvate during long-term culture in M9 minimal medium containing a high concentration (0.8%) of glucose, and the cells were later proved to be in the VBNC state (Tanda *et al.*, unpublished data). Consequently, we hypothesized that pyruvate is not only a metabolic intermediate of glycolysis but also a certain repair molecule that functions under various stress conditions (Tanda *et al.*, unpublished data).

In this study, the VBNC state of *Salmonella* Enteritidis cells was shown to be induced reproducibly by incubating the cells with H_2_O_2_. Using these VBNC cells, we examined the resuscitation effect of pyruvate and pyruvate analogues. In addition, the metabolic activities of VBNC cells, such as respiration and macromolecular synthesis (DNA, protein), were examined in the course of the resuscitation process from the VBNC state.

## Materials and Methods

### Bacterial cell culture

An environmental isolate of *S.* Enteritidis (SE) clone, SE Cl#15-1, obtained from CAF Laboratories (Fukuyama, Hiroshima, Japan; 33), was used (33) throughout this study. The bacteria were cultured overnight in 10 mL Luria-Bertani (LB) medium (Difco BD, Sparks, MD, USA) in a 50-mL tube at 37°C, with shaking at 150 strokes min^−1^ and then suspended in fresh LB medium at OD_550_ of 0.05. Thereafter, they were incubated at 37°C with shaking at 150 strokes min^−1^ for 105 min until the growth had reached the mid-logarithmic phase. The precultured bacteria were harvested by centrifugation at 2,850×*g* for 20 min in a swing rotor-equipped KUBOTA 8920 centrifuge (Kubota, Tokyo, Japan) at 4°C, washed twice with 10 mL ice-cold phosphate-buffered saline without calcium (PBS (−)), pH 7.5, and finally suspended in ice-cold PBS (−) at OD_550_ of 6.30 (about 1×10^9^ cfu mL^−1^).

### Oxidative stress exposure

Precultured bacteria were suspended in a 50-mL tube containing 10 mL fresh LB medium to which 0.1–10 mM H_2_O_2_ (Wako Pure Chemical Industries, Osaka, Japan) had been added. The cells (1×10^7^ cfu mL^−1^) were then incubated at 37°C for 0–60 min, with shaking at 150 strokes min^−1^, for exposure to oxidative stress. The bacteria were harvested by centrifugation as described above, and subsequently resuspended in fresh LB medium at 1×10^7^ cells mL^−1^. After serial dilution with ice-cold PBS (−), 25 μL aliquots were plated on LB-agar plates, which were then incubated at 37°C overnight. The resultant colonies were counted to estimate viable bacterial cell number (cfu), which was used as an indicator of culturability.

### Resuscitative effect of pyruvate or pyruvate analogues in liquid culture

The H_2_O_2_-treated bacteria were harvested by centrifugation as described above, suspended in M9 medium (Difco) at 1×10^7^ cfu mL^−1^ without or with 0.3–30 mM sodium pyruvate, and then incubated at 37°C for various times up to 60 min. Bacterial growth was monitored both by the density of bacterial solution, measured at 550 nm (OD_550_) with a spectrophotometer (UV-150-02; Shimadzu, Kyoto, Japan), and by cfu.

Besides pyruvate, pyruvate analogues, such as bromopyruvate, phenylpyruvate, and α-ketobutyrate (Sigma Aldrich, St. Louis, MO, USA) at 30 mM were also evaluated for their resuscitative effects by incubating VBNC cells with each analogue at 37°C for 60 min.

### Validation of the respiratory activity of the bacteria after the resuscitation processes using confocal laser-scanning microscopy

Bacteria in M9 medium containing 0.4% (w/v) glucose were incubated with the reagents of a *Bacstain* CTC Rapid Staining Kit (Dojindo, Fukuoka, Japan). CTC is an indicator of bacterial aerobic respiration (7, 31). The following procedures were performed essentially in the dark throughout the experiment: the bacteria were left to stand for 60 min in an incubator at 37°C. They were then washed with 500 μL PBS (−) by centrifugation at 18,100×*g* for 5 min at 4°C in an angle rotor-equipped TOMY MX-160 centrifuge (Tomy Seiko, Tokyo, Japan). Finally, the resultant cell pellets were resuspended in 40 μL PBS (−) and placed in the wells of a sterile, poly-l-lysine-coated 8-well glass slide. The cells were allowed to adhere to the glass by standing the slide on ice for 30 min, and then they were fixed on ice for 30 min with 40 μL of 4% (w/v) paraformaldehyde/PBS (−). Cellular DNA was stained with 5 μL of 20 μg mL^−1^ DAPI (4′,6-diamino-2-phenylindole lactate; Sigma Aldrich), after which the slide was rinsed twice with PBS (−). The samples were embedded in Perma Fluor Mountant Medium (Thermo Scientific, Rockford, IL, USA) and then applied to the slide, which was sealed with a cover glass and thereafter examined under a confocal laser-scanning microscope (LSM510; Carl Zeiss, Oberkochen, Germany).

The viability of the bacterial cell was determined using the following formula:

Viability (%)=100×live cells (CTC/DAPI-positive cells)/whole cells (DAPI-positive cells)

### Validation of the biosynthesis of DNA and protein of SE under resuscitation

Precultured SE cells were suspended in 250 mL LB medium containing 3 mM H_2_O_2_ at 1×10^7^ cfu mL^−1^ in a 1,000 mL Erlenmeyer flask and incubated at 37°C with shaking at 150 strokes min^−1^, to cause oxidative stress. The bacteria were harvested by centrifugation as described above, and then resuspended in fresh LB medium at 1×10^8^ cells mL^−1^. Alternatively, the bacteria were resuspended to 1×10^8^ cells mL^−1^ in 0.3 mM sodium pyruvate-containing M9 medium, and then incubated at 37°C for various times up to 60 min. The SE cells undergoing resuscitation were incubated with [methyl-^3^H] thymidine (74 GBq mmol^−1^; PerkinElmer, Waltham, MA, USA) or L-[^35^S] methionine/L-[^35^S] cysteine (43.5 GBq mmol^−1^: Met, 39.8 GBq mmol^−1^: Cys, EXPRE^35^S^35^S [^35^S]-protein labeling mix; PerkinElmer) for 60 min. After incubation, 10% trichloroacetic acid (TCA; Wako Pure Chemical Industries) was added to each sample with sudden chilling on ice to stop the incorporation of the radiolabel and to extract and remove the acid-soluble materials. The samples were centrifuged at 16,100×*g* for 5 min at 4°C in an angle rotor-equipped Eppendorf 5415R centrifuge (Eppendorf, Hamburg, Germany), and the resultant precipitates were washed 3 times with 5% TCA/PBS (−) by centrifugation. Finally, the acid-insoluble material in the precipitates was dissolved in 0.1 M NaOH, which was then neutralized with HCl, and the material was thereafter resuspended in ACS II liquid scintillation cocktail (Amersham, Piscataway, NJ, USA). Radioactivity of both [^3^H] and [^35^S] was measured using a TRI-CARB 1600TR liquid scintillation counter (Packard Instrument Company, Meridian, CT, USA).

### Statistical analysis

Statistical analysis and estimations of significance of difference between groups with comparable variances were performed by one-way analysis of variance (ANOVA).

## Results

### Reduction in culturability of SE by H_2_O_2_

**Fig. 1:**
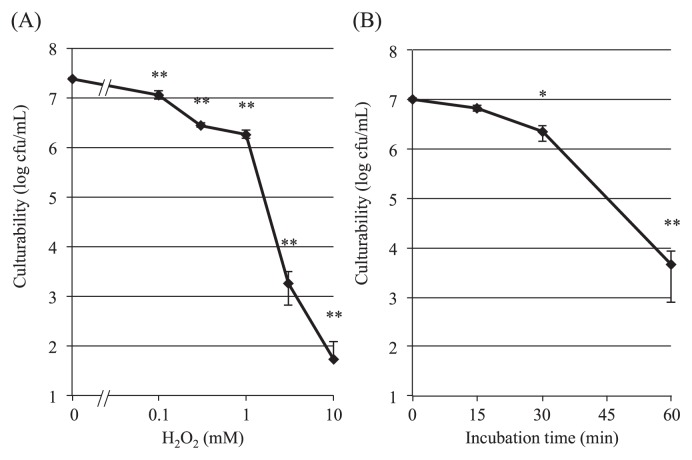
Reduction in culturability of SE cells by hydrogen peroxide (H_2_O_2_). Dose-response (A), and time-course (B) effects of H_2_O_2_ are shown. Mid-logarithmic SE cells were incubated with various concentrations of H_2_O_2_ for 60 min (A), or with 3 mM H_2_O_2_ for 60 min (B) as described in the text, and the culturability (cfu mL^−1^) was examined by plating the culture on duplicate LB agar plates. Results are shown as the mean±SEM for 3 independent experiments with significance of difference (*P<0.01 *vs.* 0 time, **P<0.001 *vs.* control (A) or 0 time (B), one-way ANOVA).

In this study, we first examined the effect of H_2_O_2_ on the culturability of SE cells. As shown in Fig. 1A, incubation of the cells with H_2_O_2_ for 60 min had a biphasic and dose-dependent effect on viability as assessed in terms of colony-forming units (cfu); Although low concentrations (0.1–1 mM) of H_2_O_2_ did not affect the culturability of SE markedly, with 1 mM H_2_O_2_ reducing the culturability by only 1/10 of that of the control, H_2_O_2_ at higher concentrations, *i.e.*, 3 and 10 mM, significantly reduced it. The cfu value decreased to approximately 1/10^4^ of the control with 3 mM H_2_O_2_ and became undetectable with 10 mM H_2_O_2_.

To determine the optimal exposure time to stress with 3 mM H_2_O_2_, we examined the time-course of incubation up to 60 min. As shown in Fig. 1B, SE cells lost their culturability in an incubation time-dependent manner, with the cfu value decreasing gradually to about 1/10 of the control by 30 min, and then rapidly to 1/2.2×10^3^ of the control by 60 min.

Based on these results, exposure to 3 mM H_2_O_2_ for 60 min was used as the standard stress condition for SE cells throughout this study.

### Restoration of culturability of H_2_O_2_-treated SE cells by pyruvate and its analogues

We next examined the ability of various chemicals to resuscitate H_2_O_2_-treated bacteria. We first tested whether pyruvate would have any effect on H_2_O_2_-treated cells, because we had earlier found that a large amount of pyruvate accumulated in the culture supernatant of SE cells after their incubation with 0.8% (w/v) glucose in M9 minimal medium, under which condition the cells had lost their culturability almost entirely but retained some metabolic activity in terms of their ability to reduce CTC (Tanda, *et al.*, unpublished data).

**Fig. 2:**
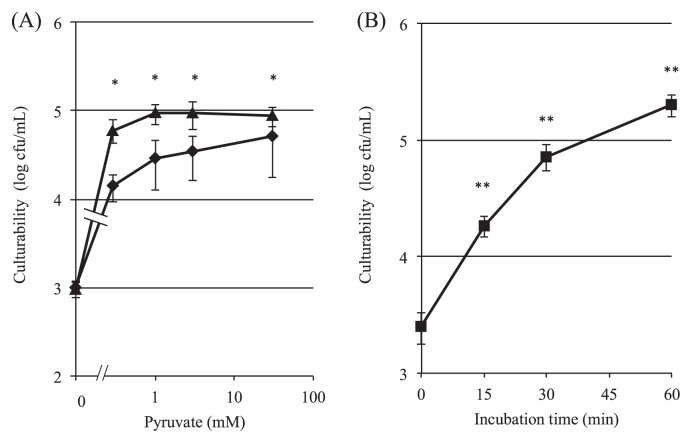
Resuscitative effect of sodium pyruvate on H_2_O_2_-treated cells in M9 minimal medium. *In (A)*, dose-response of sodium pyruvate in M9 minimal medium for resuscitation is shown, and incubation was performed for either 30 (♦) or 60 (▲) min. *In (B)*, time-course of resuscitation of H_2_O_2_-treated cells incubated with 0.3 mM pyruvate in M9 minimal medium is shown. H_2_O_2_-treated (1×10^7^ cells) cells were inoculated in 1 mL of M9 medium, and then incubation at 37°C for up to 60 min. Colony-forming activity (log cfu mL^−1^) was determined by plating cells onto LB agar in duplicate. Data are the mean±SEM of 3 independent experiments. (*P<0.05 *vs.* 0 mM pyruvate (A), **P<0.01 *vs.* 0 time (B), one-way ANOVA)

As shown in Fig. 2A, pyruvate showed a resuscitative effect on H_2_O_2_-treated SE cells in M9 medium. Incubation with pyruvate for 30 min restored the culturability of SE cells incubated by 13.8-fold to 49.1-fold, dependent by the dose of pyruvate from 0.3 to 30 mM. Longer incubation with pyruvate for 60 min also restored the culturability of SE cells, by 62.5-fold to 90.3-fold over the control (0 mM pyruvate) at concentrations between 0.3 and 30 mM. Furthermore, the addition of 0.3 mM pyruvate to M9 medium resulted in time-dependent resuscitation of H_2_O_2_-treated SE cells (Fig. 2B); however, the optical density (OD_550_) of the cultures did not increase under these conditions (data not shown). These results suggest that pyruvate increased the number of viable and culturable bacteria due to the increase of the cell population able to divide and form colonies on the LB agar plate after the resuscitation procedures. In addition, even intact bacteria (not treated with H_2_O_2_) in the logarithmic phase did not multiply in M9 medium in the presence or absence of pyruvate during 60-min incubation, as monitored by changes in both cfu and OD_550_ (data not shown).

**Fig. 3:**
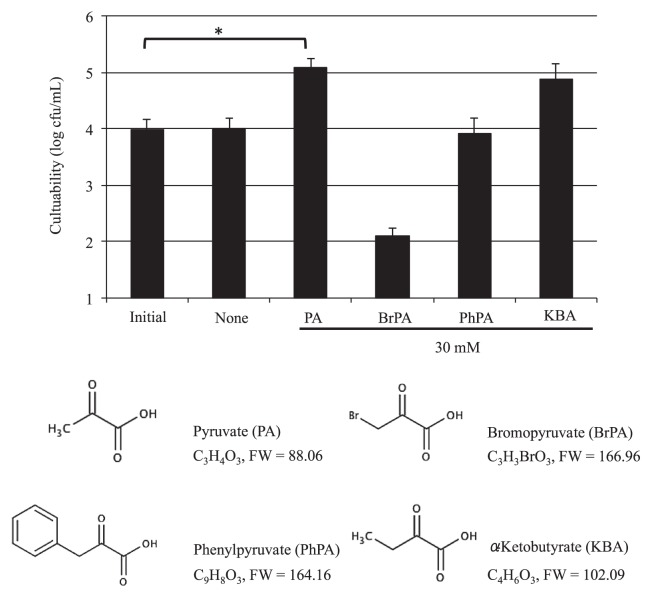
Effect of pyruvate and its analogues on the resuscitation of H_2_O_2_-treated cells. H_2_O_2_ (3 mM)-treated cells (1×10^7^ cells) were inoculated into 1 mL M9 medium containing one of the 30 mM chemicals shown on the abscissa and then incubated at 37°C for 30 min. “Initial” indicates the cfu of the cells treated with 3 mM H_2_O_2_ but subsequently with no additive and no incubation. Colony-forming activity (log cfu mL^−1^) was determined by plating cells on duplicate LB agar plates. Data are the mean±SEM of 3 independent experiments (*P<0.05, one-way ANOVA).

In addition, pyruvate analogues (bromopyruvate, phenylpyruvate, and α-ketobutyrate) were examined in this system to determine whether they would show the same resuscitative effects as pyruvate. As shown in Fig. 3, α-ketobutyrate increased cfu after incubation, although no significant difference was observed, suggesting the resuscitative effect of α-ketobutyrate other than pyruvate. In its presence, the culturability of H_2_O_2_-treated cells was restored, being 7.4-fold higher than that of the control. Phenylpyruvate had no effect, whereas bromopyruvate actually reduced the culturability of H_2_O_2_-treated cells to 1/75 of the control, probably through the inhibition of glycolysis by interruption of hexokinase II activity (18). These results suggest that the α-keto carboxy residue seems to be necessary for the resuscitation effect but that the structure of this moiety alone is inadequate to manifest the effect.

We also tested the amount of intracellular peroxide of H_2_O_2_-treated cells to avoid the possibility that pyruvate caused resuscitation by simply degrading intracellular H_2_O_2_. Quantitation of H_2_O_2_ in cell extracts of SE cells was performed with the PeroXOQuant Quantitative Peroxide Assay Kit (Thermo Scientific). As a result, there was no detectable amount of H_2_O_2_ or its adduct by the test with the assay kit, as both H_2_O_2_-treated and intact cells and the cell extract of intact SE cells degraded exogenous H_2_O_2_ (data not shown). Since α-keto acids, such as pyruvate, degrade H_2_O_2_ (12), this resuscitative effect of pyruvate and α-ketobutyrate does not seem to be simply due to its H_2_O_2_-degrading effect.

### Metabolic activities of VBNC cells before and after resuscitation with pyruvate

Under these conditions, we investigated various metabolic activities of H_2_O_2_-treated cells, including respiratory activity (measured with CTC), DNA synthesis ([^3^H]-thymidine incorporation) and protein synthesis ([^35^S]-methionine/[^35^S]-cysteine incorporation), together with culturability (colony formation).

**Fig. 4:**
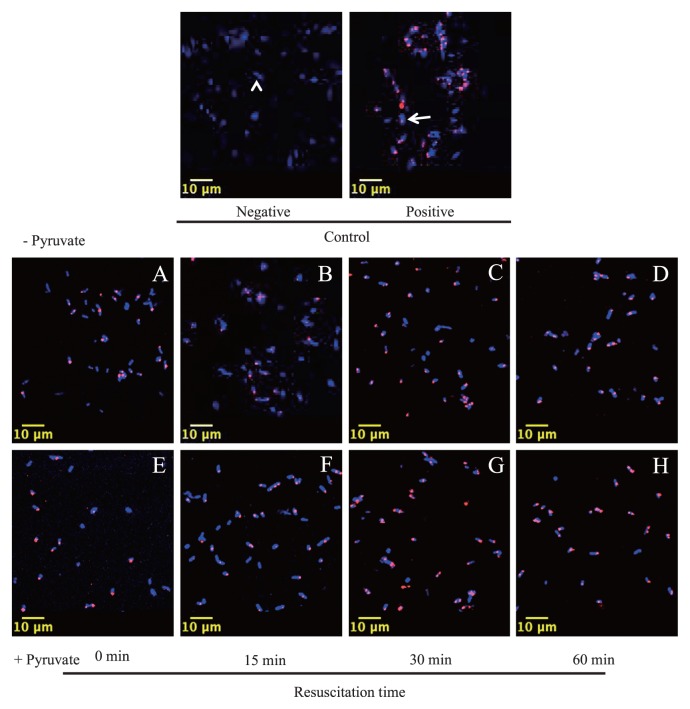
Respiratory activity of H_2_O_2_-treated cells. Confocal laser-scanning micrographs of cells stained with CTC/DAPI are shown. Viable cells with CTC-reducing activity are stained red (arrow); and total cells, stained with DAPI, are shown in blue (arrowhead) as shown in the 2 photomicrographs at the top. Dead cells correspond to cells stained with DAPI but without CTC, namely, with blue alone. The lower photomicrographs, (A) to (D) and (E) to (H), are representative images of the bacterial cells incubated without and with 0.3 mM pyruvate, respectively, after incubation for 0 (A, E), 15 (B, F), 30 (C, G) or 60 (D, H) min, as shown below the figures.

**Fig. 5:**
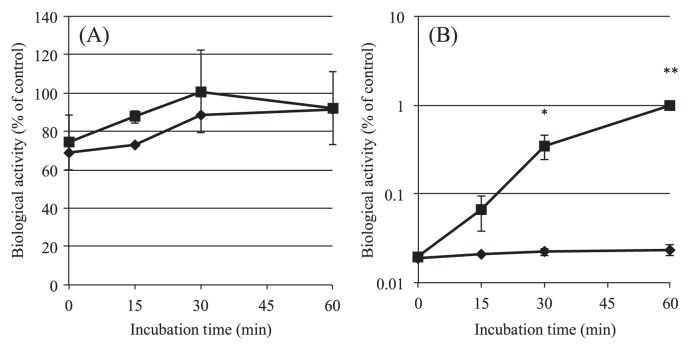
Respiratory activity of SE cells after H_2_O_2_ treatment followed by incubation without or with pyruvate. H_2_O_2_-treated cells (1×10^7^ cells) were inoculated into 1 mL M9 medium and incubated at 37°C for up to 60 min without (♦) or with (■) 0.3 mM pyruvate. The population of the cells with respiratory activity, as seen by CTC/DAPI double staining (A), as shown in Fig. 4, and the culturability (B) are shown. Each activity is shown as relative to the positive control (intact logarithmic bacteria). Data are the mean±SEM of 3 independent experiments. (*P<0.05 *vs.* 0 time, **P<0.01 *vs.* 0 time, one-way ANOVA)

As described in the Introduction, we defined VBNC cells as CTC-reduction-positive but colony-formation-negative cells in this study, and thus estimation of the metabolic activity is essential. The respiratory activity, as indicated by the CTC-reduction products seen by confocal laser-scanning microscopy, was not decreased markedly by treatment with H_2_O_2_. Many cells were positive for CTC reduction products both without (Fig. 4A–4D) and with (Fig. 4E–4H) pyruvate treatment. Based on the results of individual cell counting, H_2_O_2_-treated cells retained their activity at approximately 70% of the control (intact, non-treated; Fig. 5A), suggesting that most of these cells were in the VBNC state. In addition, activity was almost completely (92% of the control) recovered by incubation for resuscitation with or without pyruvate for 60 min, showing that the addition of pyruvate was not required for this recovery.

Contrary to the respiration activity monitored by CTC reduction, the addition of 0.3 mM pyruvate to H_2_O_2_-treated cells increased their cfu from 0.02% to 1.0% of the control, whereas the non-pyruvate-treated control remained at 0.02% (Fig. 5B). These results suggest that oxidative stress due to 3 mM H_2_O_2_ caused only moderate damage to the respiratory activity of *Salmonella*, keeping the cells in the VBNC state, and that the addition of pyruvate seemed to enable some cells to form colonies.

**Fig. 6:**
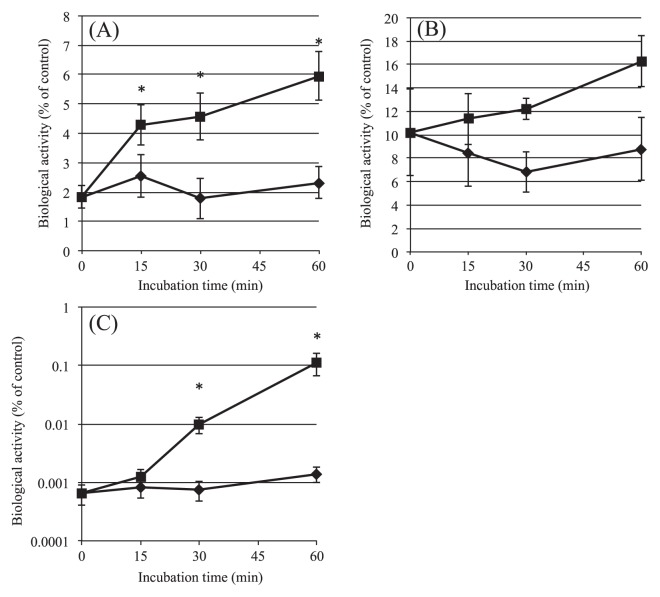
DNA and protein synthesis of SE cells after H_2_O_2_ treatment followed by subsequent incubation with pyruvate. H_2_O_2_-treated cells (1×10^8^ cells) were inoculated into 1 mL M9 medium and incubated at 37°C for up to 60 min without (♦) or with (■) 0.3 mM pyruvate. DNA synthesis, determined by performing a [methyl-^3^H]-thymidine incorporation assay (A); protein synthesis, assessed from the results of a [^35^S]-methionine/cysteine incorporation assay (B), and the culturability (C) are shown. Each metabolic activity is shown as relative to the positive control (intact logarithmic bacteria). Data are the mean±SEM of 3 independent experiments. (*P<0.05 *vs.* 0 time, one-way ANOVA)

On the other hand, unlike in the case of respiratory activity, the synthesis of protein and DNA in H_2_O_2_-treated cells was quite different in the presence and absence of pyruvate during the resuscitation process. When the cells were exposed to oxidative stress due to 3 mM H_2_O_2_, both DNA and protein synthesis decreased to 1.8% and 10.4% of the non-treated control, respectively (Fig. 6A, 6B). The addition of 0.3 mM pyruvate elevated these activities. The increase of DNA synthesis showed biphasic changes, with the synthesis rapidly increasing by 15 min, followed by a gradual increase to 3.2-fold higher than the basal level (0 time) by 60 min; however, incubation without pyruvate failed to increase it (Fig. 6A). This rapid increase of DNA synthesis seems to suggest a role for DNA synthesis in the resuscitation process induced by pyruvate.

Protein synthesis also showed a similar but moderate increase during 60 min up to 1.6-fold higher than the basal level (Fig. 6B). The culturability of H_2_O_2_-treated cells in 1,000-mL Erlenmeyer flasks was reduced to 0.0006% of the control (Fig. 6C); however, incubation with 0.3 mM pyruvate time-dependently increased culturability up to 0.11% of the non-treated control in terms of cfu. Compared with the results shown in Fig. 5B, where the culture scale was 1/10 smaller, the SE cells seemed to be exposed to much more severe stress because the cfu of 3 mM H_2_O_2_-treated cells were lower under these conditions. However, the concentration of the bacterial culture solution did not seem to affect the resuscitation activity markedly because the extent of resuscitation for 60 min in this large-scale culture (172-fold; Fig. 6C) was somewhat similar to that found for the small-scale culture (52-fold; Fig. 5B). Therefore, assessment of the activities of DNA and protein syntheses in this large-scale culture (Fig. 6A, 6B) seems to have provided comparable information for estimation of the resuscitation process.

Taken together, these results imply that some population of VBNC cells had acquired the ability to return to the culturable state by restoration of the biosynthesis of macromolecules by pyruvate, especially DNA.

In addition, we assayed the incorporation of [3-^14^C]-pyruvate into H_2_O_2_-treated and/or resuscitated SE cells. H_2_O_2_-treated SE cells incorporated the radioactive pyruvate biphasically, as they had incorporated [^3^H]-thymidine; pyruvate was rapidly incorporated by 15 min and then gradually by 60 min, similar to the case of [^3^H]-thymidine (data not shown). In keeping with the result, our study suggests that pyruvate was incorporated by VBNC bacteria, resulting in the restoration of DNA synthesis and cell division.

## Discussion

In this paper, we showed that SE cells readily changed from the growing state to the nonculturable state by brief treatment with 3 mM H_2_O_2_ during incubation in LB medium, and that these nonculturable cells could be resuscitated with pyruvate. As a result, pyruvate resuscitated the cells in liquid cultures containing M9 medium of at least 0.3 mM (Fig. 2A–B). To ascertain the chemical properties of pyruvate required for the resuscitation, we next examined pyruvate analogues, such as α-ketobutyrate, bromopyruvate, and phenylpyruvate, determining whether these analogues with α-keto carboxy residues would have a resuscitative effect on H_2_O_2_-treated SE cells. Among them, only α-ketobutyrate showed a resuscitative effect similar to that of pyruvate (Fig. 3). This analogue is converted to propionyl-CoA by oxidized ferredoxin and 2-oxobutyrate synthase (EC 1.2.7.2), and subsequently to succinyl-CoA through 3 metabolic processes, and finally integrated into the TCA cycle (15). However, our preliminary experiments suggest that activation of the TCA cycle does not seem to be essentially responsible for resuscitation, because thiamine, a cofactor of the pyruvate dehydrogenase complex that promotes the generation of acetyl-CoA, did not show any significant effect on restoration in the presence of pyruvate (data not shown). In addition, the resuscitative effect of pyruvate did not differ between experiments performed under aerobic and anaerobic conditions where, in the latter, the TCA cycle would be inactive (data not shown); therefore, the generation of energy under aerobic conditions itself does not seem to be linked to the recovery of colony-forming activity. These results seem to suggest a correlation with the reports from other laboratories showing that non-culturable bacteria, induced by low temperature and starvation, are restored to culturability by H_2_O_2_-degrading substances such as catalase, pyruvate, and α-ketoglutaric acid (6, 22–24, 30) and that non-culturable bacteria do not use these substances as nutrients, but use chemical materials with a H_2_O_2_-degrading effect, such as α-keto acid and pyruvate. However, in this study, we showed that bromopyruvate and phenylpyruvate, which possess the α-keto carboxy residue acid structure, did not restore the culturability of H_2_O_2_-treated SE cells (Fig. 3). In fact, bromopyruvate strongly decreased the number of cfu from the initial (control) level, probably through interruption of glycolysis as described above. Phenylpyruvate, which is not a metabolic inhibitor, did not change the culturability. In addition, our preliminary experiment showed that these H_2_O_2_-treated cells did not retain a detectable amount of H_2_O_2_ after H_2_O_2_ treatment. These results suggest that the resuscitative effects of α-keto acids and pyruvate are partly related to the cellular metabolism centered on the substrates around glycolysis and/or oxidative phosphorylation, not just degrading intracellular peroxide, as opposed to previous reports (6, 22–24, 30). Supporting this, α-lipoate, *N*-acetyl l-cysteine and d-mannitol, which are well-known anti-oxidant or radical-scavenging reagents, were ineffective in resuscitating H_2_O_2_-treated cells (data not shown).

The metabolic activities of the cells were decreased by H_2_O_2_ treatment (Figs. 5 and 6); however, there were large differences between culturability and the other biological markers of “viability.” Based on the definition of a “viable” state, a large population of H_2_O_2_-treated cells was viable, because 70% of the cells showed CTC reduction, and the activities were restored completely to the control levels during incubation without or with 0.3 mM sodium pyruvate due to unknown mechanisms (Fig. 5A). DNA synthesis and cell division, both of which are essential to manifest “culturability,” were restored rapidly in 15 min in the former, and gradually but greatly in the latter, only in the presence of 0.3 mM pyruvate (Figs. 5B, 6A, 6C). Protein synthesis was also restored gradually in the presence of pyruvate (Fig. 6B). These results suggest that H_2_O_2_ treatement induced a VBNC state in SE cells, and that pyruvate helped to restore DNA synthesis first and then culturability, although we could not completely rule out the possibility that a certain number of bacteria with culturability but with very low such metabolic activity in DNA or protein synthesis would gain the activity during re-incubation with pyruvate. The effect of pyruvate might not have been primarily due to any anti-oxidative effect, but to the triggering of certain metabolic activities necessary for resuscitation. Pyruvate may have activated certain metabolic pathways for energy metabolism, probably those including CTC reduction.

Further studies are required to understand the precise molecular mechanism of the resuscitation of VBNC bacteria by pyruvate and α-ketobutyrte in order to improve the risk management toward pathogenic VBNC cells threatening food safety.
